# Editorial: Precision immunotherapy and novel target discovery in hematological malignancy

**DOI:** 10.3389/fimmu.2025.1703886

**Published:** 2025-10-15

**Authors:** Beibei Zhang, Tuoen Liu, Yu Pan

**Affiliations:** ^1^ Institute of Biomedical Research, Yunnan University, Kunming, Yunnan, China; ^2^ Department of Biomedical Sciences, West Virginia School of Osteopathic Medicine, Lewisburg, WV, United States; ^3^ State Key Laboratory of Quality Research in Chinese Medicines, Macau University of Science and Technology, Macau, Macau SAR, China

**Keywords:** immunotherapy, neoantigen, target, hematological malignancy, precision medicine

Hematological malignancies, which encompass lymphoid and myeloid lineage disorders, exhibit profound molecular heterogeneity that complicates therapeutic interventions. The advent of precision immunotherapy, closely associated with the discovery of novel targets and neoantigens, has revolutionized treatment paradigms. The treatment landscape for hematological malignancies has undergone a paradigm shift with the emergence of precision immunotherapy. This approach leverages molecular insights to design interventions that target the immunosuppressive tumor microenvironment (TME) and tumor heterogeneity, which are key barriers to achieving long-lasting remission. Recent advances span novel antigen discovery, engineered cell therapies, biomarker-driven patient stratification, and combinatorial regimens that amplify immune activation while mitigating resistance.

This Research Topic highlights recent advances in precision immunotherapy and novel target discovery in hematologic malignancies. As Research Topic Editors, it was our great pleasure to curate and review a number of interesting manuscripts that covered a wide range of themes, including target discovery, immunotherapeutic engineering, and clinical translation. This Research Topic summarizes groundbreaking studies that illuminate these areas and highlight how mechanistic insights translate into clinical innovation.

(1) Novel Target Identification and Validation. Identified as an immune checkpoint upregulated in hematological malignancies, Fan et al. identified siglec15 as an immune checkpoint that is upregulated in hematological malignancies. They summarized that siglec15 remodels the tumor microenvironment (TME) by suppressing T lymphocyte activation and proliferation. This facilitates malignant cell immune escape, infiltration, and cytotoxicity, including in blood system diseases. Preclinical data support its role in immunotherapy resistance, positioning it as a promising target for immunotherapies. In acute myeloid leukemia (AML), Yan et al. reported that FLT3-ITD mutations drive immune evasion by upregulating the CD80 checkpoint via reactive oxygen species (ROS). This mechanistic link between oncogenic signaling and immune suppression suggests dual FLT3/CD80 targeting as a strategy to overcome resistance. Furthermore, Rücker-Braun et al. illustrated that HLA-B*40:01 and HLA-C*03:04 are significantly underrepresented in NPM1-mutated AML, as revealed by a large HLA association study. This was found to be closely correlated with the neoepitopes presented by these HLA alleles that trigger T-cell responses, underscoring the role of immunogenetics in defining responsive patient subgroups. In addition, Zhang et al. provided mechanistic insights into how histone deacetylases (HDACs) remodel the TME by altering chromatin accessibility, condensation, and gene transcriptional expression. These processes play pivotal roles in regulating physiological processes, cellular fate determination, and the pathogenesis of diseases, including hematological malignancy. The authors systematically elaborated on the multidimensional regulatory networks of HDACs and assessed the clinical translation progress and prospects of HDAC inhibitors (HDACis), as novel epigenetic-targeted therapeutic agents, in future precision medicine.

(2) Innovative Immunotherapeutic Platforms. CAR cell exhaustion is a major cause of relapse. Guo et al. performed an integrated analysis of anti-CD19 CAR-T cells with epigenetic modulation, and their results suggested that incorporating lysine-specific demethylase 1 (LSD1) shRNA into anti-CD19 CAR-T cells improves the efficacy against diffuse large B-cell lymphoma (DLBCL) by reducing T-cell exhaustion and prolonging persistence. In another study, Zhou et al. reported that retroviral vector-delivered anti-CD7 CAR-T cells achieved remission in refractory T-cell acute lymphoblastic leukemia (T-ALL), demonstrating the feasibility and therapeutic efficacy of this approach, along with its high safety profile, as observed in a 34-year-old male patient who developed multi-line drug resistance after undergoing high-intensity chemotherapy. In addition to T cells, natural killer (NK) cells are endowed with spontaneous cytotoxicity against infectious pathogens and cancer cells and are therefore significant in leukemia treatment. Ye et al. found that the drug aclacinomycin (ACM) sensitizes AML cells to NK cell killing by inducing increased calreticulin exposure as well as NK cell effector production of perforin and granzyme B, validating immunogenic cell death (ICD) as a bridge between chemotherapy and enhanced immunotherapy.

(3) Overcoming Resistance and Heterogeneity. Understanding leukemia-associated immunophenotypes (LAIPs) may aid in designing therapies to enhance patient outcomes. In one AML study, Gémes et al. systematically explored the multiplex immunophenotyping of AML via single-cell profiling, revealing therapy-resistant subpopulations with distinct markers. This heterogeneity underscores the need for combinatorial targeting to eliminate residual disease. This research highlights the potential of single-cell LAIP profiling and immune mediator measurements for monitoring treatment responses, identifying measurable residual disease, and detecting therapy-resistant cell populations in AML. In addition, Ye et al. demonstrated that aclacinomycin (ACM) sensitizes AML cells to NK cell-mediated cytotoxicity by enhancing the production of calreticulin, perforin and granzyme B, thus establishing immunogenic cell death (ICD) as a crucial mechanistic link between chemotherapy and augmented immunotherapy. Li et al. reported that single-nucleotide polymorphisms (SNPs) in immune-related genes impact AML treatment response and survival. The authors advocated for personalized immunogenomic screening. They found that SNPs in genes including HMOX1, TXNIP, and TNSF10/TRAIL are associated with AML and that TNFAIP2 genes serve as a critical basis for forecasting treatment responses and prognostic outcomes in AML patients. Moving to other hematological malignancies, Wang et al. conducted an integrated review to summarize and analyze the latest research advances in primary large B-cell lymphomas occurring in immune-privileged sites (IP-LBCLs), with a particular focus on emerging treatment strategies in the era of targeted therapy and immunotherapy. Moreover, in another clinical outcome observation, Wang et al. demonstrated that orelabrutinib, lenalidomide plus sintilimab achieved durable responses and a manageable safety profile in their relapsed/refractory (R/R) DLBCL patients, likely due to immune-associated TME remodeling.

(4) Clinical Translation and Biomarker-Driven Therapy. Regarding drug repurposing, Hu et al. found that fostamatinib, a SYK inhibitor used to treat chronic immune thrombocytopenia (ITP), exhibits dual cytotoxicity and immune checkpoint modulation in leukemia models, supporting its repositioning for the treatment of hematologic malignancies. Shan et al. performed analyses to identify the hub genes and immune-related pathways in AML to provide new immunotherapy theories through bioinformatics-guided target discovery. Their findings revealed Complement Factor D (CFD) as a highly expressed hub gene with a positive correlation to IL-2, which in turn exhibits positive associations with CD27 on CD24^+^CD27^+^ B cells, along with the JAK/STAT and PI3K/AKT signaling pathways. All three are positively linked to AML initiation and progression. Additional experiments were conducted to further validate and enhance the reliability of the hub gene, its physiological functions, and those of its associated immune-related pathways. In addition, Li et al. conducted an analysis combining a case report and a review of relevant literature on primary seminal vesicle diffuse large B-cell lymphoma. Their work highlights the diagnostic challenges associated with this condition and emphasizes the need for increased clinical vigilance and definitive pathological examination when managing primary seminal vesicle lymphoma. Interestingly, Liang et al. recently developed a technique that distinguishes DLBCL from CLL using Raman spectroscopy combined with bioinformatics-based identification of key genes and pathways, enabling rapid diagnostic classification. This established set of molecular markers can facilitate patient diagnosis and prognostic evaluation, providing a valuable foundation for precision therapeutic applications.

Precision immunotherapy in hematologic malignancies is rapidly evolving from target discovery to engineered solutions. By dissecting immune evasion mechanisms and leveraging cutting-edge technologies (e.g., AI-driven neoantigen prediction and precision diagnosis), recent studies provide a roadmap for next-generation therapies. Future success hinges on integrating multi-omics data, optimizing combinatorial regimens, and validating biomarkers in prospective trials.

